# Regional Delivery of Anti-PD-1 Agent for Colorectal Liver Metastases Improves Therapeutic Index and Anti-Tumor Activity

**DOI:** 10.3390/vaccines9080807

**Published:** 2021-07-21

**Authors:** Louis F. Chai, John C. Hardaway, Kara R. Heatherton, Kyle P. O’Connell, Mikayla C. Lopes, Benjamin A. Rabinowitz, Chandra C. Ghosh, Prajna Guha, David Jaroch, Bryan F. Cox, Steven C. Katz

**Affiliations:** 1 Immuno-Oncology Institute, Roger Williams Medical Center, Providence, RI 02908, USA; louisfchai@gmail.com (L.F.C.); jchardaway@gmail.com (J.C.H.); kara.heatherton@chartercare.org (K.R.H.); kyle.oconnell@chartercare.org (K.P.O.); lopesmikayla@gmail.com (M.C.L.); brabinowitz7@gmail.com (B.A.R.); chandra.ghosh@chartercare.org (C.C.G.); skatz@chartercare.org (S.C.K.); 2 Department of Surgery, Boston University Medical Center, Boston, MA 02118, USA; 3 TriSalus™ Life Sciences, Inc., Westminster, CO 80031, USA; david.jaroch@trisaluslifesci.com (D.J.); bryan.cox@trisaluslifesci.com (B.F.C.); 4 Department of Medicine, Roger Williams Medical Center, Providence, RI 02908, USA

**Keywords:** liver metastasis, regional delivery, PD-1, PD-L1, myeloid-derived suppressor cells, systemic delivery, bioluminescence

## Abstract

Metastatic liver tumors have presented challenges with the use of checkpoint inhibitors (CPIs), with only limited success. We hypothesize that regional delivery (RD) of CPIs can improve activity in the liver and minimize systemic exposure, thereby reducing immune-related adverse events (irAE). Using a murine model of colorectal cancer liver metastases (LM), we confirmed high levels of PD-L1 expression on the tumor cells and liver myeloid-derived suppressor cells (L-MDSC). In vivo, we detected improved LM response at 3 mg/kg on PTD7 via portal vein (PV) regional delivery as compared to 3 mg/kg via tail vein (TV) systemic delivery (*p* = 0.04). The minimal effective dose at PTD7 was 5 mg/kg (*p* = 0.01) via TV and 0.3 mg/kg (*p* = 0.02) via PV. We detected 6.7-fold lower circulating CPI antibody levels in the serum using the 0.3 mg/kg PV treatment compared to the 5 mg/kg TV cohort (*p* < 0.001) without increased liver toxicity. Additionally, 3 mg/kg PV treatment resulted in increased tumor cell apoptotic signaling compared to 5 mg/kg TV (*p* < 0.05). Therefore, RD of an anti-PD-1 CPI therapy for CRCLM may improve the therapeutic index by reducing the total dose required and limiting the systemic exposure. These advantages could expand CPI indications for liver tumors.

## 1. Introduction

The immuno-oncology space is evolving rapidly. Checkpoint inhibitors (CPIs) have revolutionized the treatment of certain solid tumors, including melanoma and non-small cell lung cancer. Rather than directly attacking the tumor, CPIs harness the power of the endogenous immune system by preventing the exploitation of the immune-evasive mechanisms tumors employ through the CTLA-4 and PD-1/PD-L1 pathways [1,2,3]. 

After the approval of ipilimumab for melanoma by the Food and Drug Administration (FDA) in 2011, additional CPIs such as nivolumab, atezolizumab, pembrolizumab, avelumab, durvalumab, and cemiplimab became available to an ever-growing list of indications including other cutaneous malignancies, urogenital, pulmonary, and small and large bowel carcinomas amongst others [4,5,6,7,8,9,10,11,12,13,14,15,16,17,18]. These molecules improved patient outcomes with durable tumor responses, improved progression-free survival, and increased overall survival rates in previously treatment-refractory cancers [19,20,21,22]. With the exception of successes with hepatocellular carcinoma and mismatch repair (MMR) deficient stage IV adenocarcinomas, the clinical impact of CPIs on liver tumors have been limited. 

In addition to the limited efficacy offered by CPIs in treating hepatic malignancies, immune-related adverse events (irAEs) limit the application of these potent therapies. The severity of irAEs range from mild constitutional symptoms to severe organ failure and permanent debilitating effects such as pituitary insufficiency [23,24,25,26,27]. Emergence of severe irAEs may preclude continuation of an otherwise effective therapy, which limits the potential for durable control of advanced solid tumors. This delicate balance between managing irAEs and tumor control is an ongoing challenge facing clinicians, and minimizing the frequency of side effects can only improve the impact of CPIs further. 

To overcome this barrier, we developed a preclinical model of murine colorectal cancer liver metastasis (CRCLM) to study a regional delivery (RD) technique. This has the benefit of limiting systemic exposure, thereby avoiding uptake in non-target tissues. We hypothesized that RD of CPIs would improve the therapeutic index compared to systemic delivery (SD) by enhancing intrahepatic effect while limiting extra-hepatic exposure. 

## 2. Materials and Methods

### 2.1. Animals

Six to ten-week-old C57BL/6 J male mice were purchased from Jackson Laboratories (Bar Harbor, ME). All mice were housed under pathogen-free conditions in Roger Williams Medical Center (RWMC). Procedures and animal handling and care were carried out based on an experimental protocol reviewed and approved by the RWMC Institutional Animal Care and Use Committee (IACUC) using standard guidelines and under the supervision of a licensed veterinarian.

### 2.2. Murine CRCLM Model

C57BL/6 J mice were anesthetized using aerosolized isoflurane (3–5%, Patterson Veterinary, Devens, MA, USA) and 2.5 × 10^6^ MC38-CEA cells with a luciferase reporter protein (MC38-CEA-luc) were delivered via splenic injection to generate CRCLM. This was followed by splenectomy to confine tumor growth to the liver. Post-operatively, buprenorphine (0.05–0.1 mg/kg) or buprenorphine SR (0.5–1 mg/kg) (Patterson Veterinary, Devens, MA) was injected subcutaneously for analgesia and treated with SQ 0.9% saline. 

### 2.3. Bioluminescence Monitoring and Quantification

Anesthetized mice received 100 μL of XenoLight D-Luciferin via intraperitoneal (IP) injection and were placed individually in the IVIS machine to monitor the tumor progression. Each mouse was imaged three days after tumor inoculation to establish baseline tumor burden before treatment and subsequently on each post-treatment day (PTD). Tumor bioluminescence (TB) was quantified as total flux (protons/s) using LivingImage 4.7.2 software (PerkinElmer, Waltham, MA) with values that were normalized to the baseline (PTD0) bioluminescence value (photons/s). TB < 1.0 × 10^5^ photons/s at PTD0 was considered as background and, thus, mice needed a TB of >1.0 × 10^5^ photons/s for inclusion in the study. 

### 2.4. CPI Delivery

Tumor-bearing mice were treated with 0.3 mg/kg, 1.0 mg/kg, 3.0 mg/kg, or 5.0 mg/kg of a Rat IgG2a Isotype anti-mouse PD-1 antibody (Clone: RMP1-14, Bio X Cell, Lebanon, NH) via the portal vein (PV) for RD or tail vein (TV) for SD. Mice treated with phosphate buffered saline (PBS) via PV served as vehicle control. Doses were selected based on the standard weight-based dosing used in human trials. For PV delivery, a sterile catheter composed of polyurethane tubing (0.017in ID × 0.037in OD) attached to a 30G access needle (Nipro, Bridgewater, NJ, USA) was connected to a 25G blunt tipped needle (Instech Laboratories, Inc., Plymouth Meeting, PA, USA) and 10 mL syringe (BD, Franklin Lakes, NJ) for infusion. The syringe was placed in an automated pressure injector (Harvard Apparatus, Holliston, MA, USA) and desired volumes were set accordingly. 

Once the injection catheter was prepared, an exploratory laparotomy was performed on tumor-bearing mice. The PV was cannulated with the 30G needle and advanced until just proximal to the bifurcation of the right and left hepatic branches with the liver retracted cranially. The access needle was removed following the delivery of the doses by maintaining manual pressure over the insertion site for hemostasis. Next, the organs were replaced in their anatomic positions and the fascia was closed with 4-0 Vicryl (Ethicon, Somerville, NJ, USA) followed by skin clips for the skin. The mice in the TV cohort were anesthetized as mentioned above and placed in a restraint chamber with tails submerged in warmed water to dilate the lateral tail veins. Once sufficient dilation was achieved, a 30G ½” needle was attached to a 1 mL syringe (BD, Franklin Lakes, NJ, USA) and therapy was delivered. Analgesia and fluid replacement were provided post-operatively and all mice placed in a warmed chamber. 

### 2.5. Cell Isolation

Liver non-parenchymal cell (L-NPC) isolation was performed as previously described with several modifications [28]. Mice were euthanized via terminal cardiac puncture, and, immediately following, the liver was explanted and a portion of the tissue was placed directly into a gentleMACS™ C tube with RPMI 1640 and enzymes from the Tissue Dissociation Kit for mechanical disruption with the gentleMACS™ dissociator (Miltenyi Biotec, Bergisch Gladbach, Germany). Samples were incubated at 37 °C for 40 min prior to the second round of dissociation and the resulting cell suspension was washed through a 70 μm MACS SmartStrainer (Miltenyi Biotec, Bergisch Gladbach, Germany) with RPMI 1640. Hepatocytes were separated via low-speed centrifugation followed by density gradient separation using 40% Optiprep (Sigma-Aldrich, St. Louis, MO, USA) and Gey’s Balanced Salt Solution (Sigma-Aldrich, St. Louis, MO, USA). The remaining cells were ACK lysed (Gibco by Life Technologies, Grand Island, NY, USA) and blocked with 1 μg of anti-FcγR III/II mAb2.4G2 (Miltenyi Biotec, Bergisch Gladbach, Germany). CD45^+^ cells were isolated by using CD45 immuno-magnetic beads (Miltenyi Biotec, Bergisch Gladbach, Germany) to obtain L-NPC without liver sinusoidal endothelial cells (LSEC). In general, 30% CD11b^+^ L-MDSC are present in CD45+ cells. Isolated cells were stained immediately for flow cytometry or cryopreserved for later studies.

### 2.6. Flow Cytometry and Antibodies

Isolated L-MDSC and tumor cells were stained with antibodies specific for murine CD11b, Ly6C, Ly6G, PD-L1, and human CD66 to assess MDSC and tumor phenotypic expression of PD-L1. Unstained cells and isotype controls were used for setting laser voltages. Results were analyzed with FlowJo 10.6.1 (Tree Star Inc., Ashland, OR, USA) and gating performed using isotype controls. 

### 2.7. Serum Studies 

Mice were sacrificed via terminal cardiac puncture. Collected blood was allowed to coagulate for 4–6 h at 4 °C. Serum was separated from the blood by spinning in a microcentrifuge for 10 min at 2000 rcf and the serum was transferred to a 1.5 mL Eppendorf tube, diluted with deionized water to a total volume of 200 μL, and sent to the RWMC clinical laboratory for complete metabolic panel and bilirubin analysis. For analysis of serum cytokines, serum was diluted 2-fold and analyzed by Beckman Coulter (BC; Brea, CA, USA) AU system using BC AU system ALT, AST, and Bilirubin reagents according to the manufacturer’s protocol. Data were analyzed using REMISOL Middleware software (BC, Brea, CA, USA). Remaining serum was used for enzyme-linked immunosorbent assay (ELISA) or cryopreserved. A sandwich ELISA was performed to detect anti-PD1 antibody (ThermoFisher, Waltham, MA, USA) according to the manufacturer’s protocol. 

### 2.8. Western Blots

Tumors were washed twice with ice-cold PBS and lysed with RIPA buffer (Life Technologies, Carlsbad, CA, USA) supplemented with protease inhibitor cocktail (Roche Diagnostics, Basel, Switzerland), as described previously [29]. Protein was quantified by using Bradford protein assay (ThermoFisher, Waltham, MA, USA). Lysates were denatured using Laemmli sample buffer (Bio-Rad, Hercules, CA, USA). The immunoblots were analyzed and quantified using ImageJ software. Antibodies to Ki-67 (SolA15; eBiosciences, San Diego, CA, USA), PD-L1 (B7-H1; R&D Systems, Minneapolis, MN, USA), PD-1, cleaved caspase 9, and GAPDH (D7D5W, D3Z2G, and D4C6R; Cell Signaling Technology, Danvers, MA, USA) were used at a 1:500 dilution.

### 2.9. Statistics

Statistical analysis was performed using Prism 8 (GraphPad Software, San Diego, CA, USA). Data are displayed as mean ± standard error of the mean (SEM) with corresponding values of n. Statistical significance was calculated using the students’ *t*-test and ANOVA. Values with *p* ≤ 0.05 were determined to be significant. Group-based Grubbs’ test was performed on bioluminescence to mathematically identify outliers which were excluded from the study. Using both criteria, *n* = 1–2 animals were excluded from analysis in each of the eight groups uniformly.

## 3. Results

### 3.1. Liver Metastases Promote Immunosuppression in the Tumor Microenvironment via the PD-1/PD-L1 Axis

**Figure 1:**
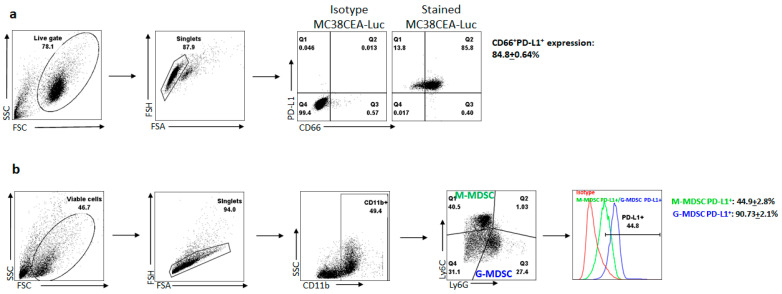
PD-L1 expression on tumor cells and MDSCs. (**a**) Gating strategy of PD-L1 expression on MC38-CEA tumor cells. Isotype controls were used for PD-L1 and CD66 for setting gates. After doublet cell exclusion, co-staining with CD66 (CEA) and PD-L1 antibodies showed high expression of PD-L1 on tumor cells. Evaluation of expression was performed in biological replicates (*n* = 3). (**b**) Gating strategy of PD-L1 expression on G- and M-MDSCs. Isotype controls were used for setting gates. After doublet cell exclusion, G-MDSC was identified as CD11b^+^Ly6G^hi^Ly6C^lo^ and M-MDSC was identified as CD11b^+^Ly6G^lo^Ly6C^hi^ phenotypes, respectively. Green box denotes M-MDSC and blue box denoted G-MDSC. Evaluation of expression was performed in biological replicates (*n* = 4).

Previous publications by our group have detailed the high levels of PD-1 expression of tumor-infiltrating lymphocytes (TILs) within the solid tumor microenvironment (TME) [30]. When bound by PD-L1, a signaling cascade results in profound immunosuppression, limiting the tumor-killing ability of the TILs. We hypothesized that CRCLM cells would mediate immunosuppression through the PD-1/PD-L1 axis and create a TME that further exacerbated this. To confirm expression levels of PD-L1 in the liver TME, we examined tumor and suppressor cell expression of this protein. After 48 h in culture, 84.8 ± 0.64% of MC38-CEA-luc cells expressed PD-L1 (Figure 1a). We confirmed the high expression of 90.73 ± 2.1% of PD-L1 in granulocytic MDSC (G-MDSC) and 44.9 ± 2.8% in monocytic MDSC (M-MDSC) (Figure 1b). 

### 3.2. Anti-PD-1 Antibody Effective as In Vivo Checkpoint Inhibitor Therapy against Tumors

**Figure 2:**
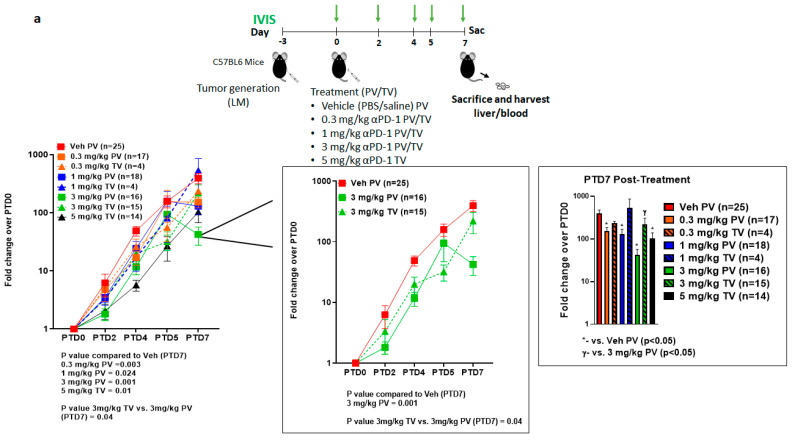

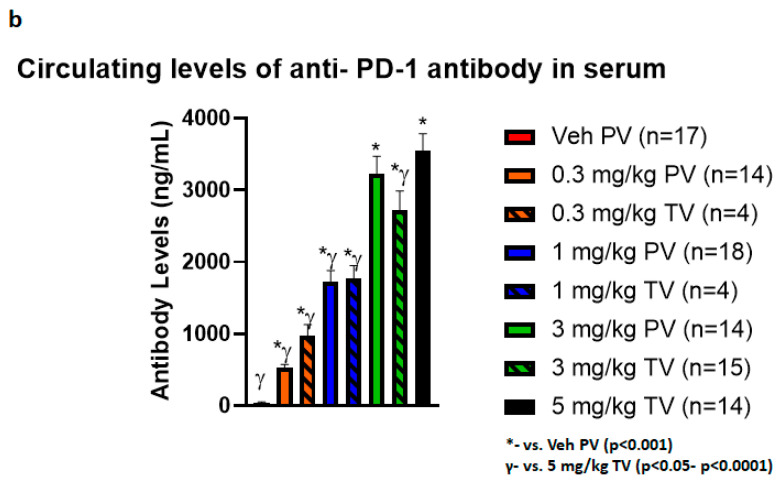
Regional delivery of anti-PD-1 treatment inhibited tumor growth at 3 mg/kg dose. (**a**) Schematic representation of tumor development with MC38-CEA-luc and treatment timeline. Mice were separated into eight treatment groups and treated according to the schema depicted with vehicle control (Veh) via portal vein (PV) or 0.3 mg/kg, 1 mg/kg, 3 mg/kg tail vein (TV) or PV and 5 mg/kg TV. Number of mice for each group is shown in the graphs. Bioluminescence (green arrows) was measured using IVIS imaging on post-treatment day (PTD)0 (baseline), PTD2, PTD4, PTD5, and PTD7 and represented as fold over PTD0 in log scale. Right graph shows the inset of Veh PV, 3 mg/kg PV, and 3 mg/kg TV and PTD7 bioluminescence comparison of different doses and routes of administration. Results are shown as mean ± SEM. (**b**) Circulating levels of anti-PD-1 antibody were assessed on PTD7 to determine levels of systemic exposure in the serum via a sandwich ELISA against Rat IgG2a proteins. The 0.3 mg/kg PV dose demonstrated significantly lower circulating levels against all other doses regardless of route of delivery while the 1.0 mg/kg PV dose also showed significantly reduced amounts compared to higher doses regardless of route of delivery. There was no significant difference seen between circulating levels of antibody when comparing the 3.0 mg/kg dose directly between PV and TV. Furthermore, 3.0 mg/kg TV did show statistically significant levels lower than 5.0 mg/kg TV, but this did not translate into appreciable differences in efficacy.

We previously reported that L-MDSC inhibits CAR-T-dependent tumor cytotoxicity in vitro, which gets reversed by targeting STAT3 that induces apoptosis in L-MDSC [31,32]. To investigate whether a similar effect in TME is observed with anti-PD-1 treatment, we challenged mice with intra-splenic MC38-CEA-luc to generate LM followed by treatment on day 3 with varying concentrations (0.3–5 mg/kg) of anti-PD-1 treatment delivered via TV or PV as shown in Figure 2a. Our in vivo results revealed improved response at 3 mg/kg on PTD7 via PV as compared to 3 mg/kg via TV (*p* = 0.04) and compared to vehicle control (*p* = 0.001) (Figure 2a). Significant differences were seen in TB for all escalating doses delivered via PV compared to vehicle control and only in 5 mg/kg TV compared to vehicle control (PTD7 *p* < 0.05). The minimal effective dose at PTD7 was 5 mg/kg (*p* = 0.01) via TV and 0.3 mg/kg (*p* = 0.02) via PV compared to vehicle control indicating a lower dose requirement via RD to achieve similar anti-tumor activity as observed with SD. No significant difference between delivery routes, PV or TV, was observed for any time point for any of the lower doses (0.3 mg/kg, 1 mg/kg). 

### 3.3. Reduction in Systemic Exposure with Low Dose Regional Delivery

Using an ELISA assay, the serum of treated mice was assessed for levels of circulating anti-PD-1 antibody and all doses were detectable compared to the vehicle control cohort (*p* < 0.001 for all comparisons, Figure 2b). This study revealed that serum levels of the anti-PD-1 agent increased in a dose-dependent fashion, regardless of route of delivery. When comparing similar doses, there were no significant differences found between 3 mg/kg PV vs. 3 mg/kg TV (3233.10 vs. 2714.09 ng/mL, *p* = 0.17). However, the lower RD doses at 0.3 mg/kg PV and 1 mg/kg PV both resulted in significantly less detected antibody in circulation compared to all higher doses regardless of route of delivery (0.3 mg/kg PV, *p* < 0.001 for all comparisons, 1.0 mg/kg PV, *p* < 0.01–*p* < 0.001). There was a 6.7-fold reduction in comparing 0.3 mg/kg PV to the minimum efficacious systemic dose of 5 mg/kg TV (*p* < 0.001). Additionally, the 3 mg/kg TV cohort revealed lower amounts of antibody than 5 mg/kg TV (*p* = 0.03), indicating an increase in circulating anti-PD-1 antibody does not necessarily correlate to improved response (Figure 2a). 

### 3.4. Equivalent Hepatic Toxicity Comparing Delivery Methods with Similar Doses

**Figure 3:**
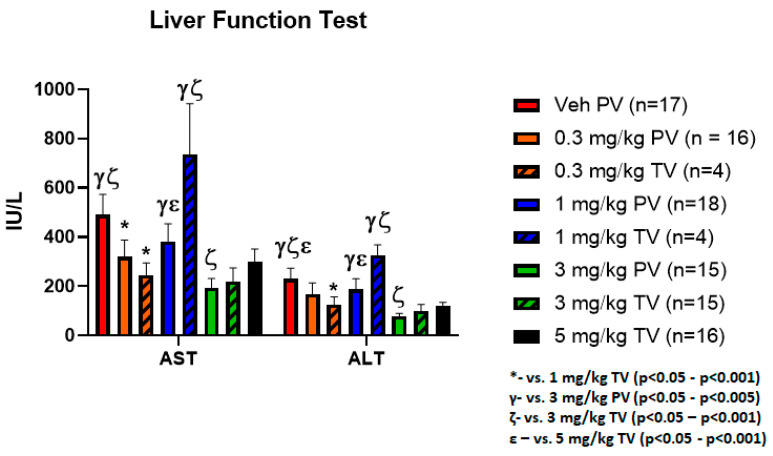
Anti-PD-1 treatment caused no significant increase in liver toxicity as compared to vehicle control. Serum assessment for hepatic toxicities was performed by measuring AST and ALT. Significant difference was seen on ANOVA analysis between the treatment groups, though this is likely a result of the elevated LFTs caused by tissue damage by the tumor in the vehicle control and 1.0 mg/kg TV groups rather than administration of the anti-PD-1 antibody. The elevations in these two groups suggests that the LFT elevation is a result of increased tumor burden when correlated with the bioluminescence data.

Serum was assessed for liver toxicities by performing liver function tests (LFT) including aspartate transaminase (AST) and alanine transaminase (ALT) of each treatment group. Significant differences were seen in LFTs when performing ANOVA analysis across all groups (AST *p* = 0.005, ALT *p* = 0.004, Figure 3). This is likely influenced by the elevations in AST and ALT seen in the vehicle control and 1 mg/kg TV cohorts, which when examined with the bioluminescence data is suggestive of hepatic damage secondary to tumor burden rather than toxicity from treatment. The equivalent AST and ALT levels when comparing higher dose SD and the RD doses also supports this conclusion while indicating that RD techniques do not appreciably result in additional liver tissue damage. Importantly, there was no significant increase in AST or ALT due to the administered doses via PV or TV as compared to vehicle control, indicating no anti-PD-1 related liver toxicity.

### 3.5. PD-1 Inhibition Promotes Apoptotic Signaling in Tumor

**Figure 4:**
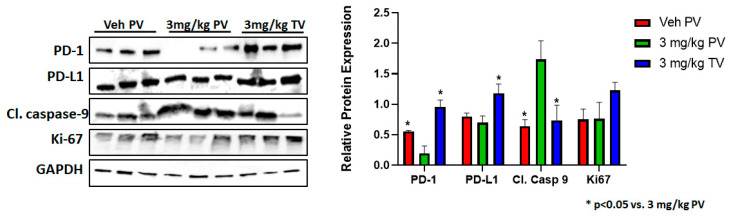
PD-1 blockade inhibits tumor growth by decreasing proliferation and increasing apoptosis in TME. Tumor lysates from vehicle control, 3 mg/kg PV and 3 mg/kg TV were analyzed by Western blot with antibodies against PD-1, PD-L1, cleaved caspase-9, Ki-67. GAPDH was used as a loading control. Triplicate samples were loaded (*n* = 3 mice/group) and the signals were quantified using densitometric analysis and normalized with GAPDH protein expression. Results are shown as mean ± SEM.

To evaluate the apoptosis/proliferation of tumor cells due to PD-1 inhibition, LM tumor lysates from PTD3 were isolated from three mice that received 3 mg/kg via either TV or PV, and vehicle control groups, respectively. These groups were selected to allow a direct comparison between delivery routes using the same doses. Western blot analysis showed significantly low PD-1 protein expression in 3 mg/kg PV as compared to vehicle control (*p* < 0.05) which confirmed increased inhibition of PD-1 in PV as compared to TV or vehicle control with no change in PD-L1 expression in the tumors (Figure 4). Interestingly, the level of cleaved caspase 9 in 3 mg/kg PV was induced, suggesting increased apoptosis as compared to vehicle control and 3 mg/kg TV groups (*p* < 0.05). There was also a decreasing trend, though not significant in Ki67 expression in 3 mg/kg PV as compared to 3 mg/kg TV and vehicle control. Raw data of western blots that were used for Figure 4 with quantification for individual bands for each protein is available in Supplementary Figure S1.

## 4. Discussion

CPIs have revolutionized the management of solid tumors. Since the first use of ipilimumab for metastatic melanoma in 2011, both the number of drugs and the diversification of applications have dramatically increased [4,5,6,7,8,9,10,11,12,13,14,15,16,17,18,25]. This class of therapy functions to inhibit checkpoint molecules within the solid TME, one of the potent immune-evasive mechanisms utilized by tumors to evade immunity [1,33,34,35,36,37]. Through use of CPI therapy, the endogenous immune system can function through tumor recognition and upregulation of antigen-specific responses. We confirmed here previously published data showing high levels of PD-L1 on MC38-CEA-luc tumor cells, and L-MDSC in the TME supports the use of this model for our studies [28].

CPI-related autoimmune-like toxicities such as colitis, dermatitis, and hepatitis are caused by SD of the antibodies that activate immune response in both tumor lesions and healthy organs. RD techniques have been used to increase delivery, overcome physiologic barriers at the target site, and increase effective drug concentration. The pharmacologic rationale and advantage for this approach over traditional SD is to avoid the uptake of therapy throughout the rest of the body before reaching the target site [38]. Applications have included peripheral cutaneous malignancies, peritoneal malignancies, pancreatic ductal adenocarcinomas, and CRCLM, with methods ranging from isolated limb perfusions to organ-specific vascular delivery [39,40,41,42,43,44,45,46,47,48,49,50,51]. When applied appropriately, meaningful clinical outcomes have been seen with improved therapeutic efficacy and tumor control. In line with this, our experimental model produced CRCLMs that had enhanced control when RD was employed with similar efficacy to higher dose SD.

With regards to achieving sufficient therapeutic concentrations, multiple dose-range finding studies have investigated optimal CPI dosing [52,53,54,55,56,57,58,59]. Both preclinical models and clinical trials have demonstrated that there is no meaningful increase in efficacy between doses with over 100-fold differences and that lower doses already saturate the majority of the relevant receptors up to 100% [60,61,62,63,64,65,66,67]. The data indicate that lower doses may be equally as effective, especially when targeted appropriately using techniques such as RD. Our data support these hypotheses as RD results in similar efficacy even with over 10-fold lower concentration to the minimum effective systemic dose up to one week after treatment. 

Variation of biological effect is dependent on which ligand, PD-L1 or PD-L2, binds to PD-1. One model shows reverse roles of PD-L1 and PD-L2 signaling in activation of natural killer T cells [68]. Inhibition of PD-L2 leads to enhanced T helper 2 cell activity, while PD-L1 binding to CD80 has been shown to inhibit T-cell responses [69]. Blocking PD-1 facilitates inhibition of signaling via both PD-L1 and PD-L2 axis. From a toxicity standpoint, irAEs directly associated with CPI therapy have presented novel clinical challenges. Despite the incredible successes in the solid tumor arena, CPIs has been associated with an alarmingly high frequency of irAEs with studies and reviews reporting incidences including all grades up to 85%, though this is variable depending on specific therapies and clinical applications [2,23,27]. A likely explanation for the high incidence of irAEs is the non-specific binding of CPIs to naturally occurring receptors present throughout the body that normally serve to regulate against self-antigen recognition, activation, and autoimmunity [70,71,72,73]. When standard infusion of CPI therapy via SD occurs, there are high levels of systemic exposure, increasing the risk of irAEs. RD strategies avoid these undesirable effects by directing therapy to the target site while maintaining therapeutic efficacy. Additionally, PD-1 detection decreased following 3 mg/kg PV showed compared to 3 mg/kg TV, possible due to neutralization of PD-1 in the liver TME. Increase PD-1 engagement following PV infusion may have further caused an increase in tumor cell apoptosis that enabled lower effective doses with RD. 

Limitations of our study include the absence of complete responses CPI treatment. While the RD groups and high-dose SD groups helped to slow tumor progression relative to vehicle control, there remained a persistent increase in tumor burden. A number of factors likely contribute to this, but we speculate that the elevated interstitial fluid pressures (IFPs) associated with solid tumors plays a significant role in this. The distorted architecture and lymphovascular disarray results in pressures much higher than mean circulating pressures, resulting in the inability of immune cells to enter the TME [74]. Though we were able to disrupt the PD-1/PD-L1 axis with respect to tumor cell engagement, the activated natural immune cells may have still been unable penetrate the stroma to act against the tumor. In order to achieve control of tumor growth and eradicate disease, we believe a combinatorial approach with CPIs may prove beneficial [14,75]. The FDA approved PD-1 inhibitor, pembrolizumab (KEYTRUDA, Merck & Co., Inc., Kenilworth, NJ, USA), on 16 June 2020 for the treatment of solid tumors with high tumor mutational burden (TMB). Studies show correlation of TMB with response to anti-PD-1 therapies, supported by a pooled analysis of 27 tumor types [76]. It is unclear whether TMB itself or the resulting influx of activated T cells accounts for the higher response rates to CPI. If the latter, combinatorial approaches may enable deeper CPI responsiveness through induction of more permissive or “hot” tumor microenvironments.

Additionally, a complete dose-range finding study was not performed, particularly with regards to the RD cohort where no minimum effective concentration was found. Doses below 0.3 mg/kg may have resulted in similar therapeutic efficacy and control of CRCLM while further reducing systemic exposure, both of which are additional benefits worthy of further exploration with lower dosing. Finally, though liver toxicity and clinical status of the mice did not appear to be impacted by the dosing or delivery route of the anti-PD-1 antibody, further examination of extra-hepatic autoimmune effects would be warranted given reported cases in the clinical arena. However, preclinical mouse models of CPI therapy with solid tumors do not always recapitulate the human response and are generally more resistant to development of irAEs and careful study design to assess these variables must be considered to augment our experiments [70,77,78,79,80]. In summary, we show that RD of anti-PD-1 antibody can overcome the SD related autoimmune toxicities and provide comparable anti-tumor efficacy with over 10-fold lower concentration as compared to the minimum effective systemic dose.

## 5. Conclusions

Applications for CPI therapies have rapidly increased in the treatment of solid tumors, with some indications as potential first or second-line options. However, the use of CPIs has been associated with high frequencies of irAEs related to dosing and delivery routes. RD of CPI represents a viable option to address both these issues as our model has demonstrated similar therapeutic efficacy using these techniques with significantly lower doses and systemic exposure. We believe that these data can be translational and support using regional delivery to improve outcomes and reduce side effects for CPI therapy. 

## Data Availability

Data is contained within the article or Supplementary Material.

## Supplementary Materials

The following are available online at https://www.mdpi.com/article/10.3390/vaccines9080807/s1, Figure S1: Raw data of Western Blots.

## Abbreviations

ALT	Alanine transaminase
AST	Aspartate transaminase
CPI	Checkpoint inhibitor
CRCLM	Colorectal cancer liver metastases
ELISA	Enzyme-linked immunosorbent assay
FDA	Food and Drug Administration
G-MDSC	Granulocytic myeloid-derived suppressor cells
IACUC	Institutional Animal Care and Use Committee
IP	Intraperitoneal
irAE	Immune-related adverse events
LFT	Liver function tests
L-MDSC	Liver myeloid-derived suppressor cells
L-NPC	Liver non-parenchymal cells
MMR	Mismatch repair
M-MDSC	Monocytic myeloid-derived suppressor cells
PBS	Phosphate buffered saline
PTD	Post-treatment day
PV	Portal vein
RD	Regional delivery
RWMC	Roger Williams Medical Center
SD	Systemic delivery
SEM	Standard error of mean
TB	Tumor bioluminescence
TILs	Tumor infiltrating lymphocytes
TMB	Tumor mutational burden
TME	Tumor microenvironment
TV	Tail vein
