# Supplementary material for: Regional Delivery of Anti-PD-1 Agent for Colorectal Liver Metastases Improves Therapeutic Index and Anti-Tumor Activity
Source: Vaccines (Basel). 2021 Jul 21;9(8):807. doi: 10.3390/vaccines9080807 (PMC8402391; doi:10.3390/vaccines9080807)
Supplement: Supplementary file 1 [file vaccines-09-00807-s001.zip › vaccines-1229384-supplementary.pdf]

Supplementary Figure S1: Raw data of Western Blots

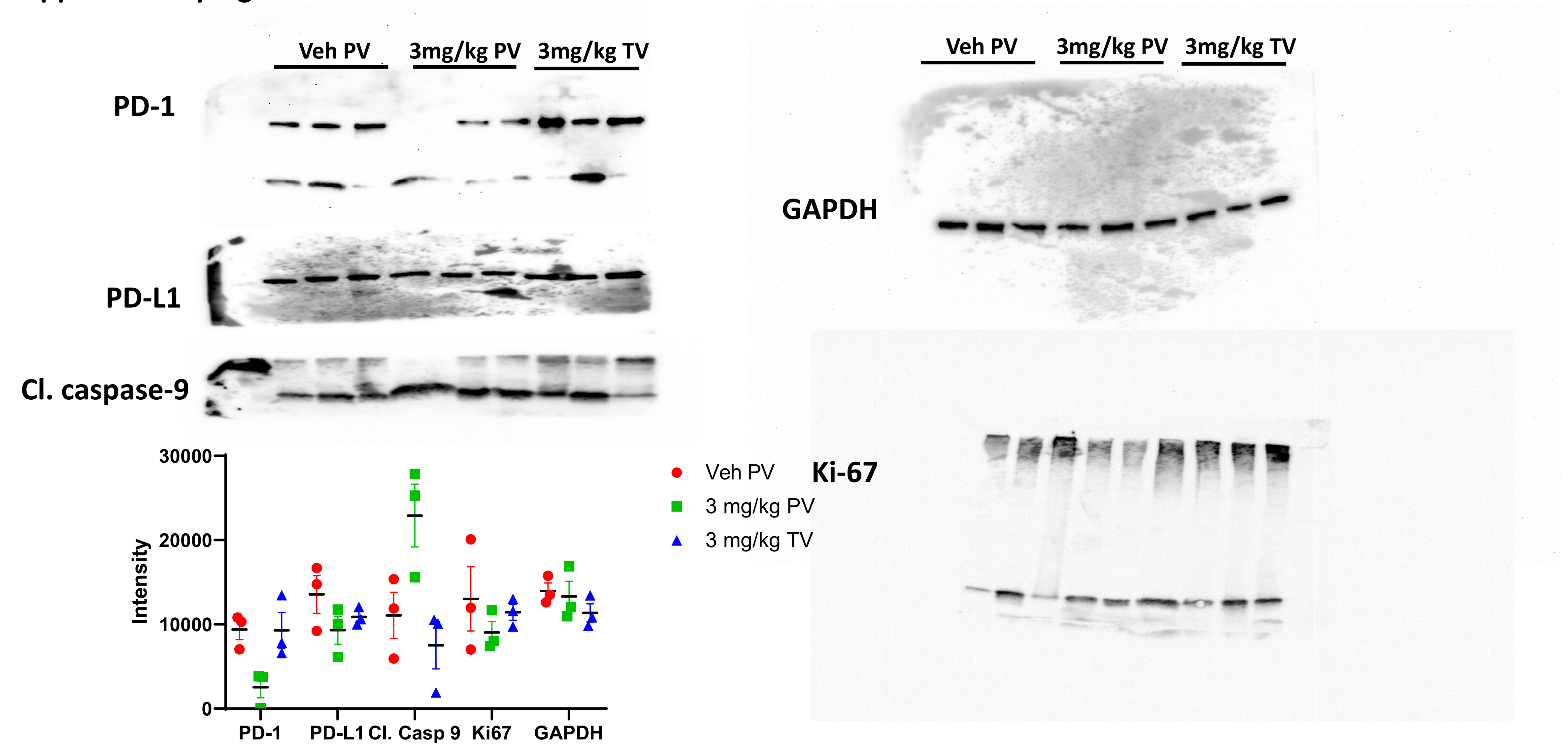

Western blot of reduced protein lysates detected for PD-1, PD-L1, Cleaved caspase-9, Ki-67 and GAPDH. Intensity of individual bands were quantified using ImageJ software. Individual gel band intensity for each protein was plotted as shown in the graph.
